# The Impact of Biosecurity on Biological and Psychosocial Risks for Health Workers of COVID Hospitals in Guadalajara, Jalisco, Mexico

**DOI:** 10.3390/ijerph20010858

**Published:** 2023-01-03

**Authors:** María de la Luz Galván-Ramírez, María de Lourdes Preciado-Serrano, Mildred Gallegos-Bonifaz

**Affiliations:** 1 Department of Microbiology Pathology, Centro Universitario de Ciencias de la Salud Universidad de Guadalajara, Sierra Mojada 950 Colonia Independencia, Guadalajara 44340, Mexico; 2 Department of Public Health, Centro Universitario de Ciencias de la Salud Universidad de Guadalajara, Sierra Mojada 950 Colonia Independencia, Guadalajara 44340, Mexico

**Keywords:** COVID-19, SARS-CoV-2, health care workers (HCWs), biological risk, psychosocial risk, hospitals, fatigue, anxiety, depression

## Abstract

Background: Some data support that health care workers (HCWs) must have sufficient and good quality personal protective equipment (PPE) and the necessary training to manage COVID patients to avoid contagion that can lead to death. The objective of this study was to determine the relationship between biosafety on the biological risks of SARS-CoV-2 and risks of fatigue, anxiety, or depression in health workers who care for patients in COVID hospitals, from September 2020 to August 2021. Material and methods: The questionnaire used in this study (Q6S64I) consisted of 6 spheres: Sociodemographic aspects, working conditions; Personal Protection Equipment; safety and health; training and knowledge about COVID-19, the form of transport, and personal health conditions. The answers were online. The Goldberg questionnaire (EADG) measures anxiety and depression, and the questionnaire measures fatigue (Barrientos-Gutiérrez et al.) (PSSF). Results: In total, 76.5% of the HCWs were doctors, 25.2% worked in the emergency services, 79.3% received PPE from their institution, 82.9% cared for COVID-19 patients, and 27.9% tested positive for COVID-19. The PPE provided by the employer was 80%, but the quality was deficient, insufficient, and associated with a relative risk of 4.6. A total of 99% acquired better PPE on their own. The exposure to COVID-19 and the surgical mask provided by the institution had an associated relative risk of 2.8 for the HCWs. A total of 39% of the HCWs reported being calm. Conclusions: PPE, risk exposure, and safety at work were significantly associated with drowsiness and heaviness, difficulty concentrating, anxiety, and depression.

## 1. Introduction

The SARS/CoV-2 coronavirus is the causal agent of COVID-19 diseases; the first report was in December 2019 in Wuhan, China. This virus has caused the largest pandemic in history [1,2]. Health care workers (HCWs) have been severely affected during the pandemic; various studies have reported many infected and deceased HCWs [1,3,4,5,6,7]. Mexico has ranked first worldwide, with the highest number of infected HCWs and deaths from COVID-19. The first report of a deceased HCWs was on March 18, 2020. 

One study analyzed two groups of HCWs; 85,477 workers classified in COVID-19 teams and 283,884 in other activities. COVID-19 team HCWs 17,186 (20%) were confirmed with COVID-19 tests, 1610 were hospitalized and 42 died. Of the 283,884 HCWs attending other activities, 38,915 (13.7%) were confirmed, 3826 were hospitalized, and 333 died [8]. In Mexico, a report of COVID-19 in HCWs confirmed 3829 infected, 97,632 treated cases, and 1320 deaths by COVID-19 [9]. From 1 January to 30 June 2021, there were 172,085 suspected cases of COVID-19 in HCWs and 43,232 confirmed cases. In all of the reports, Mexico City was in first place for infected or deceased HCWs [10].

*Biosafety.* In the COVID-19 pandemic, good biosafety practices are essential for the entire population. However, carrying them out promptly in health workers who care for patients with COVID-19 is essential due to the direct exposure and the risk of becoming infected with the SARS-CoV-2 virus. One of the biosafety actions in the attention of these patients is the use of adequate personal protective equipment and acceptable quality, as well as hand hygiene and proper management of infectious biological waste generated in the workplace. A good level of biosafety in hospitals helps to protect health personnel from becoming infected and suffering COVID-19 disease. The actions and biosafety regulations have been published and must be known and complied with for safety [11,12].

**Figure 1:**
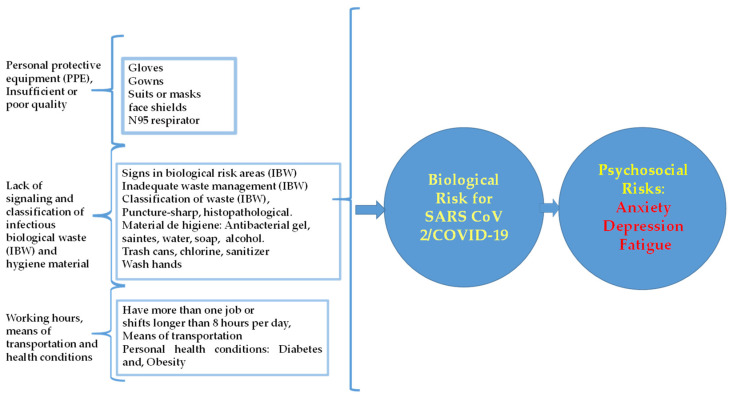
Occupational factors that promote biological and psychosocial risks in health workers in COVID-19 hospitals.

*Personal protection equipment (PPE).* The containment barriers are primary and secondary. The primary are personal protective equipment (PPE), such as gloves, gowns, suits or masks, and face shields. The secondary barriers are from isolated work areas to sinks or ventilation systems [11]; all are of the utmost importance, and the correct use of personal protective equipment (PPE) is essential to prevent contagion [12,13,14,15,16]. Studies exist that demonstrate the importance of N95 respirators [17,18]. Another study demonstrated the importance of using PPE (gloves, masks, and glasses) and that it is indispensable to decreasing the spread of COVID-19 among HCWS [7] (Figure 1).

*Working conditions, and safety in (TS).* The regulations on the disposal of biological-infectious waste establish that these must be disposed of strictly, following the specific procedures based on their type [12]. Risk communication is also mandatory in workplaces at the international level. In Mexico, the identification and communication of biological risks for handling infectious agents are in NOM-087-ECOL-SSA1-2002 [12,13]. On the other hand, workers must receive all the protective equipment from their employers and have working conditions with all the implements and biosafety actions. These are mandatory to be granted by the employer according to the International Labor Organization (ILO) and federal labor law (LFT) in Mexico [14,15,16].

On the other hand, severe symptoms of depression, anxiety, insomnia, distress, and compassion fatigue have been found in HCWs. These symptoms were more severe in women, and doctors’ compassion fatigue was more frequent [17,18,19]. According to some authors, “COVID-19 cases can lead to the collapse of the health system, and therefore, HCWs would be under stress” [18,19].

Psychosocial risks (exposure to risk, training, knowledge, and individual health conditions) due to COVID-19 exist for HCWs. According to NOM-035-STPS-2018, occupational psychosocial factors include dangerous and unsafe conditions in the work environment, such as workloads when they exceeded the worker’s capacity, and lack of control over work, long working hours, and shift rotation, including night-shift without recovery and rest periods. Interference in the work-family relationship, poor leadership, and negative connections at work can cause fatigue, depression or anxiety disorders, a non-organic sleep-wake cycle, and severe stress and adaptation (Figure 1) [20].

The HCWs have physical and mental exhaustion from the COVID-19 pandemic. Thus, the crisis has caused them stress, anxiety, depressive symptoms, insomnia, denial, anger, and fear [21]. Reports exist that these factors can compromise their mental and physical health [22,23,24]. Another study demonstrated how these symptoms affect the quality of care, clinical understanding, and the decision-making skills of COVID-19 patients [25,26,27,28].

Comorbidities have complications in the development of COVID-19. A study conducted in China and others in the UK found that infected HCWs had comorbidities [23,24,28]. In Mexico, there are reports that comorbidities increase the risk of the evolution of COVID-19 and the leading causes are obesity and diabetes [8,9]. Among the symptoms that occur in COVID-19 disease are: respiratory symptoms: dyspnea, cough, odynophagia, rhinorrhea, and anosmia as well as non-respiratory symptoms: fever, headache, myalgia, diarrhea, nausea, and fatigue [23,29].

The purpose of this study was to determine the relationship between biosafety (PPE, exposure to risk, and safety at work) and psychosocial risk factors (working conditions, training and knowledge, and individual health conditions) with fatigue, anxiety, or depression in HCWs of COVID-19 hospitals in Guadalajara, during the period 2021–2022. 

## 2. Materials and Methods

### 2.1. Sample

A total of 111 workers were included from hospitals designated for COVID-19 care in the Guadalajara metropolitan area. The sample size was obtained using the following formula for cross-sectional studies, where the universe or population to be studied is unknown:

*Z*^2^*_α_* _=_ Control error α of the sample 95%:1.96, *p* _=_ Expected prevalence of workers with COVID-19 _=_ 20%, *q* = Expected Prevalence Difference = 80%, *d* = Sample error control = 8%, *n* = Sample calculation = 96 samples.

(1) $$n=\frac{Z_{a}{}^{2}\times p\times q}{d^{2}}$$

**Figure 2:**
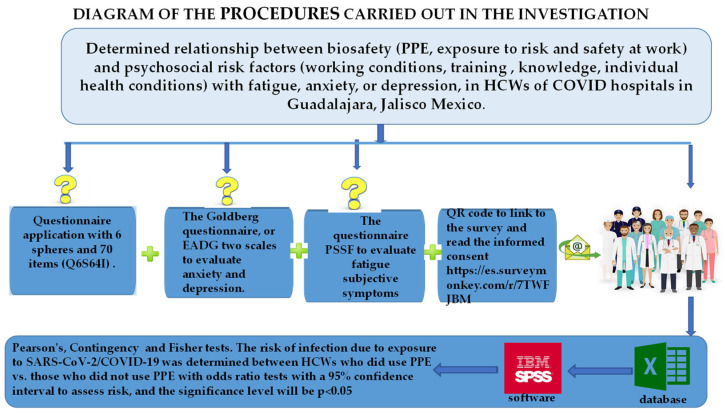
Show the procedure to determine the relationship between biosafety (PPE, exposure to risk, and safety at work) and psychosocial risk factors (working conditions, training, knowledge, individual health conditions) with fatigue, anxiety, or depression in HCWs of COVID-19 hospitals in Guadalajara, Jalisco Mexico.

This work’s complete procedure is in summary form in the flowchart of Figure 2.

### 2.2. Inclusion Criteria

Doctors, nurses, stretcher-bearers, laboratory personnel, and study cabinet, such as X-rays, social workers, psychologists, security, cleaning, and kitchen and administration personnel who have been working in the institution since January 2020 and have not presented a psychological diagnosis pre-pandemic were included.

### 2.3. Procedure

The questionnaire used in this study (Q6S64I) consisted of 6 spheres and 70 questions, described below in Figure 2. They are as follows.

Sphere 1. Sociodemographic aspects, such as age and sex and type of work they performed, Sphere 2. Working Conditions, Sphere 3. Personal Protection Equipment (PPE), Sphere 4. Safety and health, Sphere 5. Training and Knowledge about COVID-19, and Sphere 6. Transport and personal conditions.

The questionnaire was online by email, where a link was sent, or by publishing the QR code to the link to the survey and reading the informed consent https://es.surveymonkey.com/r/7TWFJBM, (accessed on 3 May 2021). Later in the Survey Monkey program, general research law protected the database [30,31].

The Goldberg questionnaire, or EADG (Spanish version, 1993) [30], were used, which consists of two scales, anxiety and depression, with nine items each, all of them with a dichotomous response (yes or no), the cut-off points were ≥4 for the anxiety scale and ≥2 for the depression scale, with a sensitivity of 74% and a specificity of 93%. The questionnaire to evaluate fatigue subjective symptoms PSSF [31], had three spheres; drowsiness and heaviness, difficulty concentrating, and projection of physical discomfort, each with ten items; all of them had a dichotomous response, the cut-off point included seven positive responses or more. The symptoms that occur in COVID-19 disease include: respiratory symptoms: dyspnea, cough, odynophagia, rhinorrhea, and anosmia as well as non-respiratory symptoms: fever, headache, myalgia, diarrhea, nausea, and fatigue [29]. In another study, the five most common symptoms among infected HCWs were: fever, fatigue, cough, sore throat, and myalgia 50 [23]. Biological and psychosocial risks are confirmed. The purpose of this study was to determine the relationship between biosafety (PPE, exposure to risk, and safety at work) and psychosocial risk factors (working conditions, training and knowledge, and individual health conditions) with fatigue, anxiety, or depression, in HCWs of COVID-19 hospitals in Guadalajara, during the period 2021–2022.

### 2.4. Statistical Analysis

Data analysis was performed with SPSS version 27 software (IBM, Los Angeles, CA, USA), and Pearson’s r test was used for parametric statistics for hypothesis testing. Contingency tables for sociodemographic variables, risk factors between infected and non-infected workers, and statistical significance were done with Pearson’s Chi-square test or Fisher’s exact probability. The infection risk due to exposure to SARS-CoV-2/COVID-19 between HCWs who used PPE and those who did not use PPE was calculated with odds ratio tests with a 95% confidence interval to assess risk, and the significance level was *p* < 0.05.

## 3. Results

### 3.1. Characteristics Sociodemographic and Working Conditions

The HCWs had an average of 36 ± years 8.49, with a minimum value of 25 years and a maximum of 70 years; 60.4% were female and 39.6% male. The occupation of 76.5% corresponds to doctors 85 (76.5%), nurses 12 (10.8%), medical assistants 5 (12.7%), cleaning staff 5 (2.7%), and others 4 (1.8%).

Regarding the institutions in which they worked: 27 (24.3%) were in private hospitals and 84 (75.7%) were in public institutions. Regarding the working day, 83 (74.7%) worked 40 h/week, and 28 (25.3%) had days of up to 60 h. A total of 57 (51.3%) had 1 to 5 years working, the type of contract in 57 (68.2%) was definitive, while 32 (28.8%) were temporary. The distribution in hospital services was: emergency room 28 (25.2%), outpatient clinic 13 (11.7%), family medicine 6 (5.4%), internal medicine 5 (4.5%) and occupational medicine 4 (3.6%), surgery 4 (3.6%), intensive care unit 3 (2.7%) and other services 32 (28.8%), and nephrology 3 (2.7%).

### 3.2. Personal Protective Equipment (PPE)

**Figure 3:**
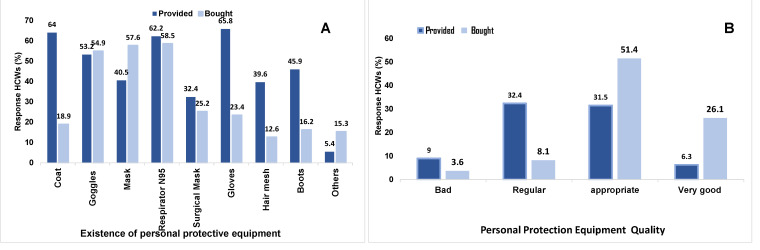
(**A**) Response of HCWs about Personal protective equipment (PPE) in COVID-19 hospitals. (**B**) PPE Quality referred by HCWs.

The protective equipment that HCWs used to work in COVID-19 hospitals, whether provided by their institution or acquired by the worker, and its quality can be seen in Figure 3.

### 3.3. Risk Exposure

**Figure 4:**
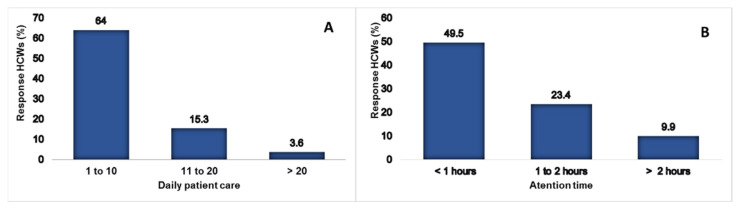
Exposure to patients with COVID-19. (**A**) Number of patients attended per shift. (**B**) Exposure time per patient.

The risk of exposure of the HCWs was analyzed considering the patients with COVID-19 they treated and the time they spent on such care, 92 (82.9%) of the HCWS cared for patients with COVID-19, and 71 (63.9%) consulted 1–10 patients/day. The majority, 55 (49.5%), did the average attention time for each patient in less than one hour. The distribution can be seen in Figure 4.

### 3.4. Hand Hygiene and Mask Use

Regarding the question about hand hygiene, 92 (82.9%) washed their hands before and after caring for patients; 91.9% reported washing their hands from 1 to 30 times per day and 8.1% more than 30 times. Concerning the number of times they changed masks, 68 (61.3%) reported that they did not alter the surgical mask/N95 respirator during the entire working day, with 15.3% doing so two times, 13.5% one time, 6.3% three times, 1.8% four times, and 0.9% 8 and 15 times.

### 3.5. Risk Signage and Disinfection Material in Different Areas at Work

**Figure 5:**
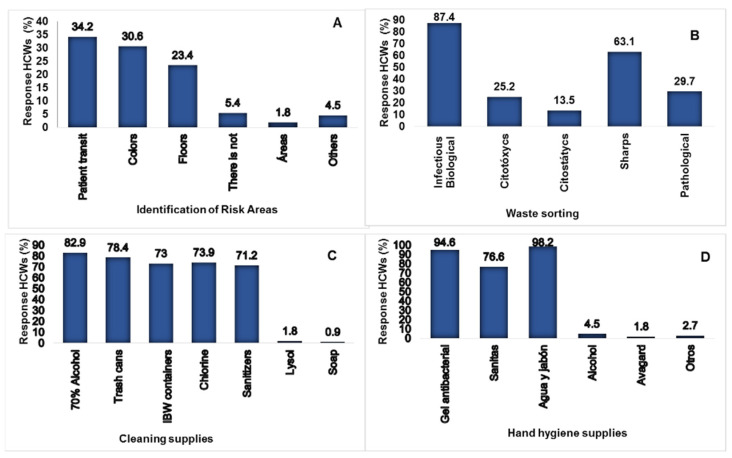
(**A**) Distribution of risk signage in different hospital areas. (**B**) Classification of hospital waste (IBW) and its type. (**C**) Materials and containers that the staff had. (**D**) Material and disinfectants that the HCWs had in their areas.

When the signage of the risk areas in the institutions was analyzed, the HCWs reported that it was adequate in 63 (56.8%) and 38 (34.2%); patient transit marks identify these infection risk areas. Regarding managing infectious biological waste (IBW), 75 (67.6%) reported correct signage. The quality of the existing material for cleaning the workplace was considered adequate in 81 (73%). According to the responses of the HCWs, the quality of the current material for hand washing and disinfection was considered suitable PPE in 73 (66%), as shown in Figure 5.

### 3.6. Training and Knowledge about SARS-CoV-2/COVID-19

**Figure 6:**
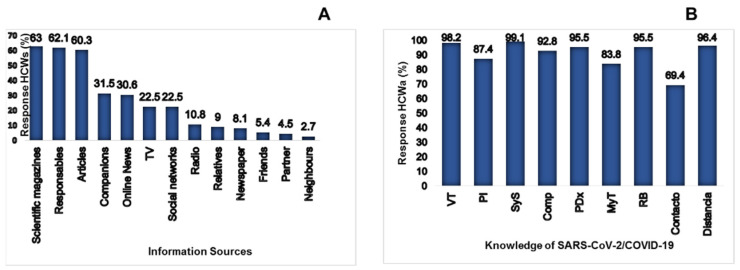
(**A**) Shows the means of information used by the HCWs to keep up to date. (**B**) Shows the knowledge that the HCWS had regarding SARS-CoV-2/COVID-19, VT: Transmission route; PI: incubation period; SyS: Signs and symptoms; Comp: Complications; PDx: diagnostic tests; MyT: Management and treatment; RB: Biological risk that it represents; Contact: Contact time with each patient and Distance: Distance you must have with the patients.

Regarding the training to care for COVID-19 patients, 87 (78.4%) of the participants received training on the management of COVID-19 patients by their institution, as shown in Figure 6. Concerning the source of information through which the HCWS obtained updates on the pandemic, 70 (63%) were kept informed through scientific journals, 69 (62.1%) by those responsible for the institution, and the rest through the information media, as shown in Figure 6B. The specific knowledge acquired covered different aspects, such as transmission routes and incubation periods; these can seen in Figure 6B.

### 3.7. Contagion Risk Level

**Figure 7:**
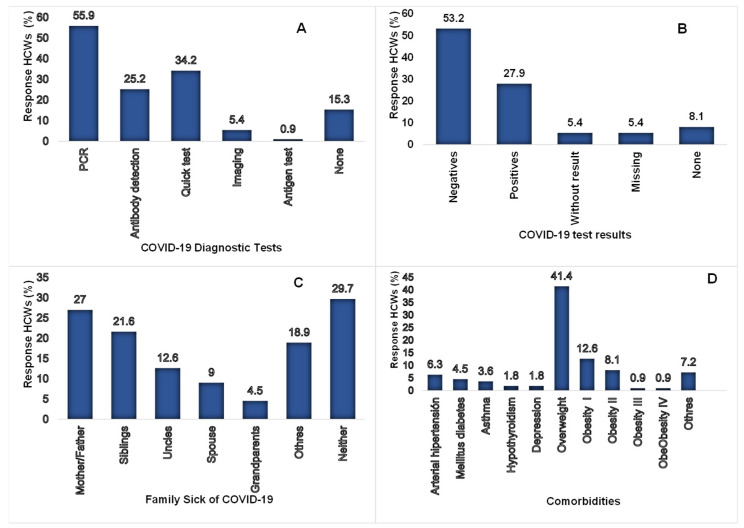
(**A**) Shows the types of tests performed on the HCWs, (**B**) results of the COVID-19 tests, (**C**) COVID-19 contagion in the family nucleus of the HCWs with whom they lived and (**D**) Comorbidities presented by the workers.

To assess the level of contagion in the HCWs, they asked about the application of COVID-19 tests; 58 (52.3%) considered it adequate and 41 (36.9%) insufficient. The type of tests carried out on the staff can be seen in Figure 7A. A total of 59 (53.2%) were negative, as shown in Figure 7B.

The most COVID-19 tests were carried out in December 2020 and January 2021, with 14 (12.6%) and 18 (16.2%), respectively. The family environment was asked to determine if there was a risk of extra-hospital transmission. Most HCWs, 78 (70.3%), had a family member sick with COVID-19; the father or mother was the most affected with 30 (27%), see Figure 7C. Of existing comorbidities in the HCWs, the primary health problem was overweight in 46 (41%) and 25 (22.5%) with some level of obesity. The distribution of comorbidities can be seen in Figure 7D.

The risk of contagion was analyzed with Pearson’s X^2^ or Fisher’s exact test, depending on the criteria. There was a statistically significant association between the COVID-19 patient care variable and the quality variable of the PPE provided by the institution with a Fisher’s exact test of *p* < 0.01. The PPE analyzed with respect to patients with COVID-19 showed a significant *p* < 0.05 relationship with the surgical mask and gloves provided at their institution.

The gloves given by the institutions and the quality of the PPE acquired by the HCWs were analyzed, and a chi-square of *p* < 0.01 is shown in Table 1. The relative risk of contagion concerning the quality of the protective equipment used and provided by the institutions was analyzed, resulting in 4.6 times more risk due to poor quality protective equipment as shown in Table 1.

No statistically significant associations were found between the PPE provided by the institution and its quality; however, between 10–30% of the participants considered such quality poor. The PPE provided by the institution with the results of the COVID-19 tests were analyzed; no was fount significant associations; however, 10–20% of the participants who had a positive test of COVID-19 did not receive the full PPE for their complete protection.

Likewise, the consultation of patients with COVID-19 and the PPE provided by the institution were analyzed and no significant associations were found; however, 25–55% of the HCWS treated patients with COVID-19 did not receive the PPE necessary for their safety.

Regarding the results of the COVID-19 tests with the symptoms that the HCWS presented, it identified that only three HCWs that were positive for COVID-19 gave any symptoms.

### 3.8. Emotional Status

When the emotional state of the HCWs was analyzed, they referred being calm 44 (39.6%), stressed 36 (32.4%), worried 33 (29.7%), confused 2 (1.8%), fearful 14 (12.6%), annoyed or angry 10 (9.0%), helpless 12 (10.8%), tired 1 (0.9%), depressive 2 (1.8%), and indifferent 1 (0.0%). The discrimination suffered by the HCWs was in different forms; 8 (7.2%) by neighbors, 5 (4.5%) by friends, 3 (2.7%) in transport, and 1 (0.9%) by patients and relatives or in stores. In comparison, 93 (83.8%) did not specify discrimination.

### 3.9. Subjective Symptoms of Fatigue Test (PSSF)

When analyzing the results of the PSSF test [31], in sphere one of drowsiness and heaviness, 33 (29.7%) presented a higher risk. While for sphere two, difficulty concentrating, a higher risk was found in 19 (17.1%), and in sphere three of projection of physical discomfort, 8 (7.2%) presented a higher risk.

### 3.10. Goldberg Anxiety and Depression Scale (EADG)

When analyzing anxiety and depression using the Goldberg [30] anxiety and depression scale, or EADG (Spanish version, 1993), 35 (31.5%) HCWs were at risk of presenting anxiety, and almost three quarters (81) were at risk of depression (73%).

The correlation of the variables studied using the Pearson test concerning fatigue, anxiety, and depression found a significant correlation of *p* < 0.05 between the variable of seniority, drowsiness, and heaviness of the fatigue test.

The number of times the surgical mask/N95 respirator was changed with tiredness and heaviness, difficulty concentrating, fatigue and anxiety through the EADG test showed a significant correlation of *p* < 0.05. Regarding the correlation between anxiety and depression, both were statistically significant with the three spheres of fatigue, with a *p* < 0.01, as shown in Table 2.

Research Hypothesis Test. To analyze the model that identified the predictor variables of the biosafety condition and psychosocial risk factors with fatigue (drowsiness-heaviness, lack of concentration, and physical discomfort), anxiety, and depression, multiple linear regression was applied with the method “Enter”. Significance was found in two dimensions of the PSSF (spheres one and two) and the dimension of depression Table 3.

The regression model for sphere 1: Drowsiness and heaviness of the fatigue variable indicated a 37% predictive value. The regression model for sphere 2: Lack of concentration of the fatigue variable stated a 32% predictive value. The regression model for depression predicted a 47% predictive value. On the other hand, the variable of care for patients with COVID-19 showed a significant relationship with the variable of depression symptoms with a chi-square of *p* < 0.01 Table 4.

## 4. Discussion

The hypothesis was verified because fewer biosafety conditions were observed, and higher psychosocial risk increased fatigue, anxiety, and depression levels in HCWs.

In the analysis of (PPE) provided by their institution, almost two thirds reported receiving it. Our results are similar to a study in which their institution provided them with the same PPE. However, it is not specified in what proportion this equipment was awarded [23]. Both results show the institutional commitment to grant a high level of protection to HCWs against infectious risks. There are other reports in which the institutions gave the HCWs only gloves, a gown, protective glasses, and a surgical mask [7]. In this study, the HCWs did not have a total level of protection since they did not have the N95 respirator. A high level of security with this N95 respirator by HCWs was demonstrated. However, the time of use of the surgical mask and N95 respirator was not analyzed [18]. In our study, 61.3% of HCWs used it; however, they did not change it during their working day.

The exposure to COVID-19 in HCWs was 82.9%; this result is higher than reported, where 59.1% cared for patients with COVID-19 [23]. On the other hand, the virulence of SARS-CoV-2 increases consultation in hospitals, leading to HCWs having a higher exposure to contagion.

Concerning hand hygiene in the HCWs, the majority, 82.9%, washed their hands before and after each consultation. Some washed their hands up to 30 times in a working day. These results are similar to the study previously reported regarding the frequency of hand washing of 491 HCWs. However, the number of times is unspecified in this study [17].

In the signage of areas of risk at work, 34.2% reported that the infection risk areas of their hospital are through traffic signs and a minority, 1.8%, in the service area. Our results are not comparable with other studies included in this work since there is only one study where it was only proposed to delimit the areas or zones of infection risk [32].

Regarding the signage on the management of IBW analyzed in the hospitals where the HCWs worked, 67.6% reported that their institution had correct signage for IBW; however, in the studies reviewed, no information was found in this regard. The preceding demonstrates the lack of knowledge or interest in other studies on managing IBW, such management is crucial to prevent the spread of infections within hospitals.

The quality of the existing material for cleaning the workplace and hand hygiene qualified by the HCWs was adequate at 73% and 66%, respectively. There was no existing information regarding these variables in our literature search. The quality of hygiene and cleaning materials is essential as a biosafety factor for HCWs, so it must considered in future studies.

According to the health of HCWs and the risk of COVID-19infection, the detection in HCWs was investigated. The main tests that were performed were PCR (55.9%), rapid test (34.2%), and antibodies (25.2%). The HCWs that had a positive result in some tests were 27.9%; this percentage was higher than in other studies [7,8,28,29], which could be due to factors such as lack of quality and training in proper use of PPE. A study demonstrated that Personal Protection Equipment availability is essential not only for professionals’ physical health but also for their mental health [33].

When analyzing the fatigue results, 29.7% presented a higher risk of drowsiness and heaviness, 17.1% difficulty concentrating, and 7.2% physical discomfort. Of the articles considered, none analyzed fatigue with the same instrument as in our study (subjective symptoms of fatigue test, PSSF) nor any other device.

Regarding anxiety, 31.5% of the HCWs were at risk of presenting anxiety; this result was higher than the percentage found in other studies [19,23,25], possibly due to the workload that HCWs had during the pandemic, increase in COVID-19 consultations, the risk of infection, and the lack of adequate PPE.

Regarding depression, almost three quarters presented a risk of depression (73%), while other studies reported 50.4% [8,23] and 65.8% in Paraguay [19]. Depression may be because, in our research, the HCWs suffered from various concerns, coupled with anxiety, fatigue, and loss of life of patients, family, and friends.

When the training of HCWs for the care of patients with COVID-19 was analyzed, 78.4% received training from their institution. These results may be due to the great need for updating and information on COVID-19 and its pandemic behavior. However, we did not find scientific references that have analyzed these variables.

The analysis of seniority in work with the variable drowsiness and heaviness found a significant correlation of *p* < 0.05, indicating that the number of years worked can increase fatigue in this area.

On the other hand, when analyzing the variable on change of surgical mask/N95 respirator with drowsiness and heaviness, difficulty concentrating, and anxiety, the three variables found significant correlations of *p* < 0.05, a result contrary to what was reported by another study [19].

The HCWs with additional work days outside the institution attending to COVID-19 patients had a higher susceptible to predicting drowsiness, heaviness, and the lack of concentration of the PSSF Test with a *p* < 0.05 and the depression of the EADG with *p* < 0.05. No statistical significance was found between the independent indicators of working hours outside the institution and care of COVID-19 patients with a projection of physical discomfort from the PSS F test and anxiety from the EADG Scale. This result may mean that having more than one job caring for patients with COVID-19 increases the workload and the risk of contagion, making them more likely to present drowsiness, lack of concentration, and depression, which has been reported in health workers caring for COVID-19 patients [34,35].

A risk of contagion was found in the care of patients with COVID-19 and the quality of the PPE; a significant association was found *p* < 0.01; surgical mask and gloves of *p* < 0.05, and depression *p* < 0.01. However, in a study, depression and anxiety in the care of patients with COVID-19 with the EPP were not significant. However, only the PPE was analyzed globally in this study, not the quality [19].

The occupation of the HCWs was 76.5% doctors and 10.8% nurses, results similar to those found previously [8,17,19,23,25,28,29,32], where the majority of the participants were nurses followed by doctors, which may be because both professions are the most frequent in the care of patients with COVID-19 within hospitals.

The HCWs worked in various hospital services, such as emergency, general wards, and outpatient clinics, with a lower proportion in family, labor, and internal medicine services, and general surgery, nephrology, and intensive care units. These results are similar to those reported [2]. The above may be because most consultations were in the emergency service, available rooms, and external consultation. This result may be due to the emergency needs caused by the pandemic, while others studies were in different services, such as oncology, hemato-oncology, urology, trauma and microsurgery, and infectious diseases, among others [18,28].

Of HCWs with a family member infected with COVID-19, 70.3% had more than one relative with this infection. These results demonstrate the importance of considering the transmission of COVID-19 from HCWs to their relatives. However, a study on 81 family members of HCWs experienced a lower infection rate than their families and did not represent the main transmission risk for relatives [36].

Regarding the eating and resting habits of the HCWs, only 53.2% had three meals a day, and 43.2% admitted sleeping six hours a day. These results reflect that almost half of the HCWs had a poor diet and little rest during the pandemic. These variables were analyzed with the presence of fatigue, anxiety, and depression in HCWs. However, no information was found in this regard despite the importance of the participants’ health.

Regarding the presence of comorbidities in the HCWs, the majority were overweight, with some level of obesity, hypertension, DM2, and asthma. Our results coincide with other studies that found that most HCWs presented hypertension, DM2, cardiovascular diseases, COPD, chronic liver disease, allergies, and immune system diseases [8,23,29,33]. These comorbidities may be due to the high prevalence of obesity and overweight in our population and poor eating habits, which can make a difference to the health of the HCWs in China and the Netherlands, where eating habits are different, and health care is of greater importance [33].

The emotional state of the HCWs was reflected as calm in 39.6%, stressed in 32.4%, and worried in 29.7%. While in other studies, HCWs reported suffering from the loss of patients and co-workers. They also reported concern about the risk of infection of colleagues and family members, protection measures, and violence against HCWs [22,27].

Regarding the presence of symptoms presented in HCWs during the pandemic, 1.8% reported fever (>38 °C), fatigue or tiredness and 0.9% nasal congestion or headache. These results were low compared to those reported in other studies. More than 50% of the participants presented fever, fatigue, myalgia or muscle pain, odynophagia, cough, tiredness, headache, and the common cold, dyspnea [7,23,29,33]. These results could be because these studies were carried out from January to May 2020, the first six months of the pandemic, so they did not know about SARS-CoV-2 and its implications and where PPE use was not optimal within hospitals.

Discrimination in HCWs for treating patients with COVID-19 was observed mainly by neighbors with 7.2%, friends, transport, relatives, in shops. The stigmatization of HCWs can be due to the general public’s fear of catching COVID-19 from HCWs. Another cause may be using the regulatory uniform by HCWs on public roads, which goes against official regulations since they must change in the same hospital before going out on the street. Although the uniform is a protection method for HCWs, it can also be a transmission factor when contaminated. This aspect is of great importance for the knowledge of good practices of health personnel. However, a study in Russia reported that the stigmatizing reactions are not directly associated with the risks of infection and are most prevalent among nurses and paramedical personnel [37].

No significant correlations were found between the variables of working hours inside and outside the institution, COVID-19 patient care, hand washing, smoking, hours of rest per day, and body mass index (BMI) with the fatigue tests (PSSF) and anxiety and depression (EADG). A similar study [19] found no significant relationship between the care of COVID-19 cases with depression and anxiety. These results can be because the HCWs were more concerned about the PPE they used to protect themselves against the risk of contagion than about the number of COVID-19 patients they treated.

Although there was no significant association between the PPE provided by the institution and its quality, the HCWs rated its quality as poor. Hence, they found it necessary to purchase PPE on their own. On the other hand, it demonstrates that some of the HCWs who had a positive result for COVID-19 did not receive the full PPE for their total protection. This result denotes the need to give more importance to the health of HCWs by the institutions where they work.

## 5. Conclusions

The actions analyzed on the biosafety level, with personal protective equipment (PPE), exposure to risk, and safety at work, were significantly associated with drowsiness and heaviness, difficulty concentrating, anxiety, and depression.

The bad quality and inadequate use of PPE are risk factors for COVID-19 in health workers.

The lack of knowledge about risk signage, the correct use of PPE, and the final disposal of infectious biological waste must be taken care of to guarantee a good level of biosafety for health care workers.

A total of 27.9% of the HCWs were infected. However, many of these could be asymptomatic, and contagion sources since 70% of all HCWs had a sick relative.

Psychosocial work conditions (workload, long working hours) were associated with drowsiness and heaviness, difficulty concentrating, and depression.

## Figures and Tables

**Table 1:** Analysis of the risk of contagion of health workers and the use of PPE in attention of COVID-19 patients.

**Quality of the protective equipment (PPE) provided by the institution**									
**Consultation COVID-19**	Bad	%	Good	%	Fisher		**RR**	ICLower	ICUpper
Yes	43	38.7	49	44	**0.01**		**4.68**	1.276	17.16
No	3	2.7	16	14.4					
**Symptoms of depression**									
**Consultation COVID-19**	Yes	%	No	%	X^2^	gl	**RR**		
Yes	19	17.1	73	65.8	11.075	0.001	**0.189**	0.067	0.536
No	11	9.9	8	7.2					
**Gloves issued by the institution**									
**Quality of (PPE) Provided by the institution**	Yes	%	No	%	X^2^	gl	**RR**		
Good	6	5.4	40	36	15.6	0	**0.155**	0.058	0.415
Bad	32	28.8	33	29.7					
**Surgical mask granted by the institution**									
**Consultation COVID-19**	Yes	%	No	%	X^2^	gl	**RR**		
Yes	66	59.5	26	23.4	4.26	0.039	**2.829**	1.029	7.732
No	9	8.1	10	9					
**Gloves issued by the institution**									
**Consultation COVID-19**	Yes	%	No	%	X^2^	gl	**RR**		
Yes	27	24.3	65	58.6	5.7	0.017	**0.302**	0.109	0.834
No	11	9.9	8	7.2					

**Table 2:** Significant relationships between the different dimensions of the instruments.

Correlations	Drowsiness and Heaviness	Difficult to Focus	Screening of Physical Discomfort	Anxiety	Depression
**CW.Labor Old**	−0.191 *	0.004	−0.118	−0.138	0.151
	**0.045**	0.967	0.220	−0.069	0.477
**WC.Working hours within the institution**	−0.002	−0.018	0.004	**0.025**	0.014
	0.982	0.852	0.966	0.793	0.887
**WC. Working hours outside the institution**	−0.269	−0.061	−0.149	0.131	−0.228
	0.118	0.728	0.394	0.453	0.188
**ER. COVID-19 patients attention per day**	0.179	0.1	0.097	0.162	0.188
	0.089	0.347	0.363	0.125	0.074
**ER.Change of surgical mask/N95 respirator**	0.190 *	0.192 *	0.1	0.228 *	0.031
	**0.047**	**0.044**	0.297	**0.017**	0.745
**ER.Hand washing/day**	0.058	0.14	0.061	0.154	0.122
	0.546	0.144	0.527	0.111	0.205
**IHC. Hours of rest/day**	−0.09	−0.08	−0.078	−0.74	−0.142
	0.352	0.404	0.421	0.071	0.14
**IHC. Cigarettes/day**	0.083	−0.003	0.116	0.093	0.117
	0.391	0.973	0.227	0.337	0.227
**IHC. Body mass index**	−0.12	−0.137	−0.041	−0.052	−0.118
	0.213	0.154	0.674	0.592	0.22
**Drowsiness and heaviness**				0.769 **	0.761 **
				**0.01**	**0.01**
**Difficult to focus**				0.809 **	0.735 **
				**0.01**	**0.01**
**Screening of physical discomfort**				0.703 **	0.688 **
				**0.01**	**0.01**

* The correlation is significant at the 0.05 level (bilateral). ** The correlation is significant at the 0.01 level (bilateral). Note: WC = Working conditions. ER = Exposure to risk. IHC = Individual Health Conditions. Numbers marked in bold = statistical significance

**Table 3:** Mathematical models for hypothesis testing.

Regression Statistics						
Model	R	R^2^	R^2^ Thigt	EEE	*p*	Equation Models
**1**	0.606	0.367	0.312	2.635	0.05	**Sphere 1** = 5.195 + (−0.148CL6.1) + 2.95CL9.3 **(Drowsiness and heaviness)**
**2**	0.512	0.316	0.256	2.252	0.013	**Sphere 2** = 3.035 + (−0.114CL6.1) + 0.223CL9.3 **(Difficult to Focus)**
**3**	0.687	0.472	0.426	2.467	0.001	**Sphere 3** = 4.566 + (−0.156CL6.1) + 0.367CL9.3 **(Depression)**

Note: CL6.1 = How many hours a week do you dedicate to your work activities outside this institution; CL9.3 = How many patients with COVID-19 do you see on average during your working day (per day)?

**Table 4:** Estimation of model 1, hours of work and average of patient’s attention per day.

Model Estimation						
**Non-standardized coefficients**				**Standardized coefficients**		
Model		B	Standard Error	Beta	*t*	*p*
1	(Constant)	5.195	0.956		5.432	0
	CL6.1	−0.148	0.048	−0.58	−3.094	0.005
	CL9.3	0.295	0.093	0.593	3.163	0.004
**Model Estimation**						
**Non-standardized coefficients**				**Standardized coefficients**		
Model		B	Standard Error	Beta	*t*	*p*
1	(Constant)	3.035	0.817		3.713	0.001
	CL6.1	−0.114	0.041	−0.54	−2.786	0.011
	CL9.3	0.223	0.080	0.545	2.794	0.10
**Model Estimation**						
**Non-standardized coefficients**				**Standardized coefficients**		
Model		B	Standard Error	Beta	*t*	*p*
1	(Constant)	4.556	0.896		5.099	0.000
	CL6.1	−0.156	0.045	−0.59	−3.468	0.002
	CL9.3	0.367	0.087	0.719	4.198	0.000

Note: CL6.1 = How many hours a week do you dedicate to your work activities outside this institution; CL9.3 = How many patients with COVID-19 do you see on average during your working day (per day)?

## Study Suggestions and Limitations

One of the study’s limitations may be the number of surveys analyzed. The contributions are aspects the biosafety such as knowledge about risk signage, the correct use of PPE, and the final disposal of infectious biological waste that are essential in new research on biosafety.
